# Lethal severe fever with thrombocytopenia syndrome virus infection causes systemic germinal centre failure and massive T cell apoptosis in cats

**DOI:** 10.3389/fmicb.2023.1333946

**Published:** 2024-01-05

**Authors:** Yusuke Sakai, Serina Mura, Yuko Kuwabara, Saya Kagimoto, Masashi Sakurai, Masahiro Morimoto, Eun-sil Park, Masayuki Shimojima, Noriyo Nagata, Yasushi Ami, Tomoki Yoshikawa, Naoko Iwata-Yoshikawa, Shuetsu Fukushi, Shumpei Watanabe, Takeshi Kurosu, Akiko Okutani, Masanobu Kimura, Koichi Imaoka, Masayuki Saijo, Shigeru Morikawa, Tadaki Suzuki, Ken Maeda

**Affiliations:** ^1^Department of Pathology, National Institute of Infectious Diseases, Tokyo, Japan; ^2^Laboratory of Veterinary Pathology, Joint Faculty of Veterinary Medicine, Yamaguchi University, Yamaguchi, Japan; ^3^Department of Veterinary Science, National Institute of Infectious Diseases, Tokyo, Japan; ^4^Department of Virology I, National Institute of Infectious Diseases, Tokyo, Japan; ^5^Management Department of Biosafety and Laboratory Animal, National Institute of Infectious Diseases, Tokyo, Japan; ^6^Faculty of Veterinary Medicine, Okayama University of Science, Ehime, Japan

**Keywords:** severe fever with thrombocytopenia syndrome, cats, histopathology, lymph nodes, germinal centre reaction

## Abstract

**Introduction:**

Severe fever with thrombocytopenia syndrome (SFTS) is a fatal viral disease characterized by high fever, thrombocytopenia, leukopenia, and multi-organ haemorrhage. Disruption of the humoral immune response and decreased lymphocyte numbers are thought to contribute to the disease severity. These findings have been obtained through the analysis of peripheral blood leukocytes in human patients, whereas analysis of lymph nodes has been limited. Thus, in this study, we characterized the germinal centre response and apoptosis in the lymph nodes of cats with fatal SFTS, because SFTS in cats well mimics the pathology of human SFTS.

**Methods:**

Lymph node tissue sections collected during necropsy from seven fatal SFTS patients and five non-SFTS cases were used for histopathological analysis. Additionally, lymph node tissue sections collected from cats with experimental infection of SFTS virus (SFTSV) were also analysed.

**Results:**

In the lymphoid follicles of cats with SFTS, a drastic decrease in Bcl6- and Ki67-positive germinal centre B cells was observed. Together, the number of T cells in the follicles was also decreased in SFTS cases. In the paracortex, a marked increase in cleaved-caspase3 positivity was observed in T cells. These changes were independent of the number of local SFTS virus-positive cell. Furthermore, the analysis of cats with experimental SFTSV infection revealed that the intrafollicular Bcl6- and CD3-positive cell numbers in cats with low anti-SFTSV antibody production were significantly lower than those in cats with high anti-SFTSV antibody production.

**Discussion:**

These results suggest that dysfunction of the humoral response in severe SFTS was caused by the loss of germinal centre formation and massive apoptosis of T cells in the lymph nodes due to systemically circulating viruses.

## Introduction

Severe fever with thrombocytopenia syndrome (SFTS) is an emerging tick-borne infectious disease caused by the bunyavirus, SFTS virus (SFTSV), which belongs to the order *Bunyavirales*, family *Phenuiviridae*, genus *Bandavirus*. This species was recently renamed *Bandavirus dabieensis* (Kuhn et al., 2023). In humans, SFTS is characterized by high fever, haemorrhagic tendencies, gastrointestinal and neuronal symptoms, thrombocytopenia, and leukopenia (Li, 2015; Kato et al., 2016; Kim et al., 2018; Saijo, 2018). SFTS is an important life-threatening disease in East Asia, with a high case fatality rate of 15 to 30% (Yu et al., 2011; Liu et al., 2013; Kato et al., 2016; Li H. et al., 2018; Kobayashi et al., 2020). Additionally, a disease similar to SFTS caused by the SFTSV-related heartland virus has been reported in North America (Fill et al., 2017; Staples et al., 2020).

Serum antibodies against nucleocapsid (N) proteins and glycoproteins play vital roles in the prognosis of SFTSV (Song et al., 2018; Wang et al., 2019). Patients with a severe disease course and deceased patients have significantly lower serum antibody levels, especially lower immunoglobulin (Ig) G levels, than patients with a mild disease course and those who survive (Song et al., 2018; Wang et al., 2019). Moreover, secondary infections with several bacterial and fungal agents due to immunosuppression have often been reported in patients with SFTS, which worsen their prognosis (Chen et al., 2018; Kawaguchi et al., 2021; Zhang et al., 2022). Thus, understanding the pathophysiology of immunosuppression and immune damage in SFTSV-infected individuals is important to improve treatment strategies for SFTS.

Analyses of peripheral blood samples from human patients have revealed a decrease in T cells and myeloid-derived dendritic cells (Sun et al., 2014; Lu et al., 2015; Liu et al., 2017; Zhang et al., 2017). However, analysis of lymph node cell populations is still lacking. This is due to the difficulty in collecting and analysing human lymph node samples. We have previously reported feline cases of SFTSV infection mimic the pathology of fatal human SFTS (Sakai et al., 2021). Furthermore, the experimental infection of cats with SFTSV reproduces severe SFTS pathology (Park et al., 2019).

Here, we analysed the lymph nodes of spontaneous cases of fatal feline SFTSV infection and found key findings that explain the severe immunosuppression in patients with SFTS, such as a defect in the germinal centre reaction and a decrease in the number of T cells due to caspase8-dependent apoptosis. The results were also confirmed by the analysis of samples from cats with experimental SFTSV infection. These results suggest that the histopathological analysis of lymph node samples is useful for studying immunological disturbances in patients with SFTS.

## Materials and methods

### Tissue samples of feline lymph nodes

Feline tissue samples with natural SFTSV infection and without SFTSV infection were collected from necropsy cases and sent to our laboratory after death for postmortem examination. In necropsy cases with clinical signs of SFTS, SFTSV infection were confirmed by conventional reverse transcription polymerase chain reaction using two primer pairs targeting the nucleoprotein region (Yoshikawa et al., 2014). The clinical information for these cases is provided in the Table 1 of our previous report (Sakai et al., 2021). The samples of systemic organs and cervical, submandibular, cervical, axillary, and mesenteric lymph nodes were collected, immersed in 10% neutral buffered formalin for fixation, and processed routinely for embedding in paraffin. The samples were subjected to histopathological experiments in agreement with the owners. Ten lymph node tissue samples from seven feline necropsy cases of fatal SFTSV infection were selected and analysed in this study. In addition, five lymph node tissues from five SFTSV-negative feline necropsy cases were analysed.

**Table 1:** List of antibodies used in this study.

Antigen	Host	Clone	Dilution	Retrieval^a^	Source
SFTSV	Rabbit	Polyclonal	1:1000	pH6.0	Gift from Dr. Shigeru Morikawa
Bcl6	Mouse	LN22	Ready to use	pH9.0	Nichirei Bioscience, Cat #418181, RRID: *AB_3073752*
Ki67	Rat	SolA15	1:1000	pH9.0	Thermo Fishcer Scientific, Cat #14–5,698-82, RRID:*AB_2865119*
Fascin	Mouse	55 k-2	1:200	pH9.0	Cell signaling technology, Cat #99978, RRID: *AB_3073767*
CD3	Rabbit	Polyclonal	Ready to use	pH6.0	Dako, Cat #IS503, RRID:*AB_2732001*
FoxP3	Rabbit	D6O8	1:200	pH9.0	Cell signaling technology, Cat #12653, RRID:*AB_2797979*
Granzyme B	Rabbit	Polyclonal	1:200	pH9.0	Abcam, Cat #ab4059, RRID:*AB_304251*
Cleaved-caspase3	Rabbit	5A1E	1:200	pH6.0	Cell signaling technology, Cat #9664, RRID:*AB_2070042*
Cleaved-caspase8	Rabbit	18C8	1:200	pH9.0	Cell signaling technology, Cat #9496, RRID:*AB_561381*
Iba1	Rabbit	Polyclonal	1:1000	pH9.0	Fujifilm-Wako, Cat #019–19,741,: RRID:*AB_839504*
FAS	Rabbit	Polyclonal	1:200	pH9.0	Santa Cruz Biotechnology, Cat #sc-1024, RRID:*AB_2100245*

a pH6.0 retrieval buffer, citrate buffer; pH9.0 retrieval buffer, histofine antigen retrieval buffer pH9.

### Grading of SFTSV positivity in lymph nodes of natural infection cases

Lymph nodes diagnosed with necrotizing lymphadenitis in natural SFTS cases were excluded because they were inappropriate for cell population analysis. Other lymph nodes from natural infection cases were graded as low or high viral load lymph nodes, based on the SFTSV N protein positivity rate of the cells. Low viral load lymph nodes contained no or only a few SFTSV-positive cells dispersed in the cortex, whereas high viral load lymph nodes contained a sizeable number of positive cell clusters in the parafollicular cortices or massive infiltration of positive cells in the cortices (Supplementary Figure S1). Cell populations were compared among five lymph nodes from non-SFTS cases and low viral load lymph nodes and high viral load lymph nodes from feline SFTS cases. Five lymph node samples from non-SFTS cases, five low viral load lymph nodes from three feline SFTS cases, and five high viral load lymph nodes from three feline SFTS cases were analysed in this study.

### Immunohistochemistry

The antibodies used in this study are listed in Table 1. Tissue sections of 4 μm thickness were de-paraffinized and heated at 121°C for 15 min in antigen retrieval buffer, as indicated in Table 1. After washing with phosphate-buffered saline (PBS), endogenous peroxidase was quenched with 3% hydrogen peroxide in PBS. After blocking with 5% skim milk in PBS for 30 min, the tissue sections were incubated at 37°C for 60 min with the primary antibodies listed in Table 1. The dilutions of each primary antibody are listed in Table 1. After washing with PBS, sections were incubated with Histofine Simple Stain MAX PO (M) (Nichirei Bioscience, Cat #424131, RRID: *AB_2811178*), Histofine Simple Stain MAX PO (R) (Nichirei Bioscience, Cat #424141, RRID: *AB_3073750*), or ImmPRESS HRP goat anti-rat IgG polymer detection kit (Vector Laboratories, Cat # MP-7404-50, RRID: *AB_ 2,336,531*). After washing with PBS, positive signals were visualized using peroxidase-diaminobenzidine reaction, and the sections were counterstained with hematoxylin.

### Double-labelling immunofluorescence

Double-labelling immunofluorescence of cleaved-caspase 3 and CD3, Iba1, or FAS was performed on 4 μm thick sections. Heat-mediated antigen retrieval was performed using an antigen retrieval solution pH 9.0 (Nichirei Bioscience). After washing with PBS and blocking with 5% skim milk, the sections were incubated with anti-CD3, anti-Iba1, or anti-FAS antibodies at 37°C for 60 min. After washing with PBS, the sections were incubated with Alexa488-conugated anti-rabbit IgG (1:400; Abcam, Cat # ab150077, RRID: *AB_2630356*) for 30 min at 37°C. After washing with PBS, the sections were incubated with Alexa555-conjugated rabbit monoclonal anti-cleaved-caspase 3 antibody (1:100, Cell Signaling Technology, Cat #9604, RRID: *AB_2797708*) overnight at 4°C. Tissue sections were incubated with 4′,6-diamidino-2-phenylindole (DAPI; 1:10,000; Dojindo, Cat #D523) for nuclear labelling. The sections were analysed using an LSM710 confocal microscope (Zeiss, RRID: *SCR_018063*).

Double-labelling immunofluorescence staining for CD3 and granzyme B was performed as follows. After antigen retrieval, blocking, incubation with an anti-CD3 primary antibody, and incubation with a horseradish peroxidase (HRP)-conjugated secondary antibody, the slides were incubated with tyramide-Opal520 (Akoya Biosciences, Cat #OP-001001) for 10 min to label the Opal520 fluorophore and heated at 95°C for 10 min to remove the antibodies from the slides. The slides were blocked again, incubated with an anti-granzyme B primary antibody and an HRP-conjugated secondary antibody, and then incubated with tyramide-Opal690 (Akoya Biosciences, Cat #OP-001006) for 10 min. The slides were then heated at 95°C for 10 min and stained with DAPI and analysed using PhenoImager fusion (Akoya Biosciences, RRID: *SCR_023274*) and processed using inForm software (Akoya Biosciences, RRID: *SCR_019155*).

### Immunohistochemistry-positive cell densities in lymphoid follicles

Lymph node tissue sections were immunostained with anti-Ki67, anti-CD3, and anti-Bcl6 antibodies. Images of five lymphoid follicles per tissue section were captured at ×200 magnification. The area of each lymphoid follicle was measured using the QuPath software (RRID: *SCR_018257*), and the number of positive cells inside each lymphoid follicle was counted. Density (number of positive cells/mm^2^) of each tissue section was calculated and averaged. Five lymph node tissue sections were analysed for each group.

### Immunohistochemistry-positive cell counts in the parafollicular region

Lymph node tissue sections were immunostained with anti-Foxp3, anti-granzyme B, anti-cleaved caspase 3, anti-cleaved caspase 8, and anti-Ki67 antibodies. Five images of the parafollicular area per tissue section were captured at ×400 magnification. The number of positive cells per high-power field (HPF) was counted and averaged for each tissue section. Five tissue sections were analysed for each group.

### Analysis of experimental infection cases

Feline tissue samples with experimental SFTSV infections were analysed in this study. The experimental protocol, clinical information, anti-SFTSV neutral antibody titres, and pathological findings of these cases have been published previously (Park et al., 2019). Briefly, six cats were intravenously inoculated with 10^7^ TCID50/mL of SFTSV SPL010 strain. Four of the six cats exhibited severe illness and were euthanized at 8- or 10-days post infection (dpi) because they met the humane endpoint. The other two cats showed no obvious clinical signs and were euthanized at 14 dpi. The systemic organs were collected, fixed in 10% buffered formalin, routinely processed, and embedded in paraffin. In this study, tissue samples from the submandibular, cervical, axillary, and mesenteric lymph nodes were analysed. The sections were immunostaind with anti-Bcl6, anti-CD3, and anti-cleaved caspase 3 and positive cell densities in the follicular or parafollicular regions were counted.

### Statistical analysis

The immunostaining-positive cell density and positive cell count per HPF data from the three groups (non-SFTS cases, low viral load, and high viral load) were first examined for normality using the Shapiro–Wilk test. For datasets with confirmed normality in all three groups, the significance between groups was examined using one-way analysis of variance (ANOVA), followed by the Tukey–Kramer test. For other datasets, the significance between groups was examined using the Kruskal-Wallis test, followed by Dunn’s test. Analyses were performed using the GraphPad Prism software (RRID: *SCR_002798*).

## Results

### Grading of lymph node tissue

As previously reported (Sakai et al., 2021), lymph nodes of cats with SFTS were histopathologically classified into three patterns: hyperplasia without SFTSV-positive cells, accumulation of SFTSV-positive cells, and necrotizing lymphadenitis. Among these three histological patterns, lymph nodes with necrotizing lymphadenitis were excluded from this study because of the massive loss of constituent cells. Lymph nodes with the other two histological patterns were graded as low or high viral load based on the SFTSV N protein immunopositivity rate of the cells. Low viral load lymph nodes contained no or only a few SFTSV-positive cells dispersed in the cortex, whereas high viral load lymph nodes contained a sizeable number of positive cell clusters in the parafollicular cortices or massive infiltration of positive cells in the cortices (Supplementary Figure S1).

### Germinal centre reaction in feline SFTS necropsy cases

**Figure 1:**
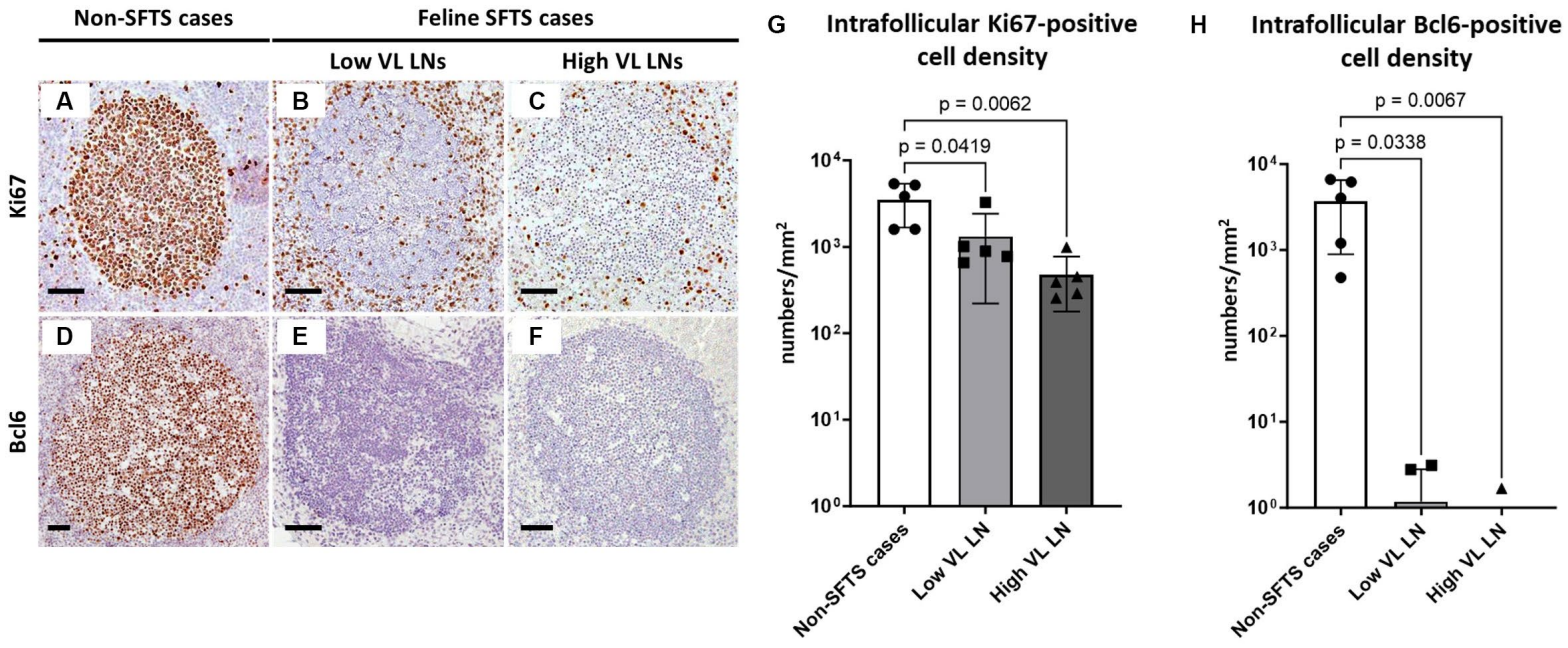
Ki67- and Bcl6-positive germinal centre B cells in the follicles of the lymph nodes of feline patients with necropsy. Representative results of immunohistochemical analysis of Ki67 **(A–C)** and Bcl6 **(D–F)**. **(A,D)** Patients with Non-severe fever with thrombocytopenia syndrome (SFTS); **(B,E)** Low viral load (VL) lymph nodes (LN), and **(C,F)** High VL LN. Bars indicate 200 μm. **(G,H)** show the density of Ki67-positive and Bcl6-positive cells, respectively, in the follicles. The bars show the average of five lymph node tissue sections and each symbol represents the density of each lymph node. Adjusted *p*-values are provided for statistically significant differences.

Based on the histopathological analysis of H&E-stained tissue sections of the lymph nodes of feline SFTS cases, we suspected a defective germinal centre response. To confirm this observation, lymph node tissue sections from SFTSV-negative and feline SFTS cases were immunostained for Ki67 and Bcl6 to label germinal centre B cells. Immunohistochemical detection of fascin, a marker of follicular dendritic cells, confirmed the preservation of follicular structures in the lymph nodes (Supplementary Figures S2A–C). Immunohistochemistry revealed a decrease in intrafollicular Ki67-positive cells in feline SFTS cases compared to that in SFTSV-negative cases (Figures 1A–C). The densities of Ki67-positive cells in low viral load and high viral load lymph nodes were approximately 50 and 15% of those in non-SFTS cases, respectively (Figure 1G). The density of Bcl6-positive cells in feline SFTS cases dramatically decreased and almost no Bcl6-positive cells were found in the lymphoid follicles (Figures 1D–F,H). These results revealed defects in germinal centre B cell activation.

### Intrafollicular T cell subpopulations in feline SFTS cases

**Figure 2:**
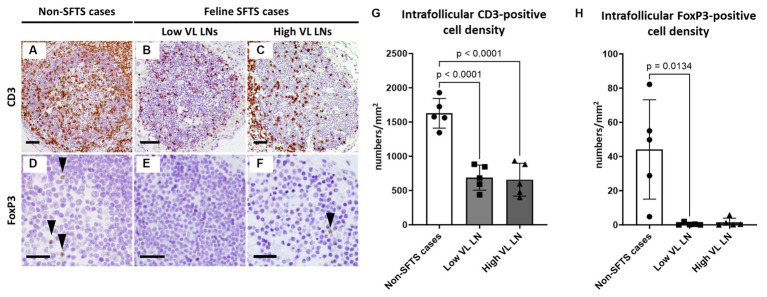
CD3- and FoxP3-positive cells in the follicles of the lymph nodes of feline patients with necropsy. Representative results of immunohistochemical analysis of CD3 **(A–C)** and FoxP3 **(D–F)**. **(A,D)** Non-severe fever with thrombocytopenia syndrome (SFTS) cases, **(B,E)** Low viral load (VL) lymph nodes (LN), and **(C,F)**: High VL LN. Bars indicate 200 μm **(A–C)** and 50 μm **(D–F)**. **(G,H)** show density of CD3-positive and FoxP3-positive cells, respectively, in the follicles. The bars show the average of five lymph node tissue sections, and each symbol represents the density of each lymph node. Adjusted *p*-values are provided for statistically significant differences.

Germinal centre reactions are regulated by various cell types including follicular stromal cells and T cell subpopulations (Stebegg et al., 2018). Follicular helper T (Tfh) cells play essential roles in germinal centre B cell activation by providing co-stimulatory molecules and secreting interleukin (IL)-21 (Vinuesa et al., 2016). Because antibodies against Tfh cell marker molecules for cats are not available, T cell numbers in the lymphoid follicles were analysed. The results demonstrated that follicular T cell numbers in feline SFTS cases were significantly decreased to less than half of those in non-SFTS cases (Figures 2A–C,G). In addition to Tfh cells, T cells that suppress the germinal centre reaction, follicular regulatory T (Tfr) cells, constitute the T cell population in lymphoid follicles (Wing et al., 2018). Thus, Tfr cells in the lymphoid follicles were analysed by immunostaining for FoxP3 (Figures 2D–F). Intrafollicular FoxP3-positive cells were rare, constituting less than 3% of the CD3-positive cells in non-SFTS cases (Figure 2H). Their densities in the SFTS cases significantly decreased and were scarcely observed (Figures 2D–F,H).

### Apoptosis and proliferation activity of paracortical lymphocytes

**Figure 3:**
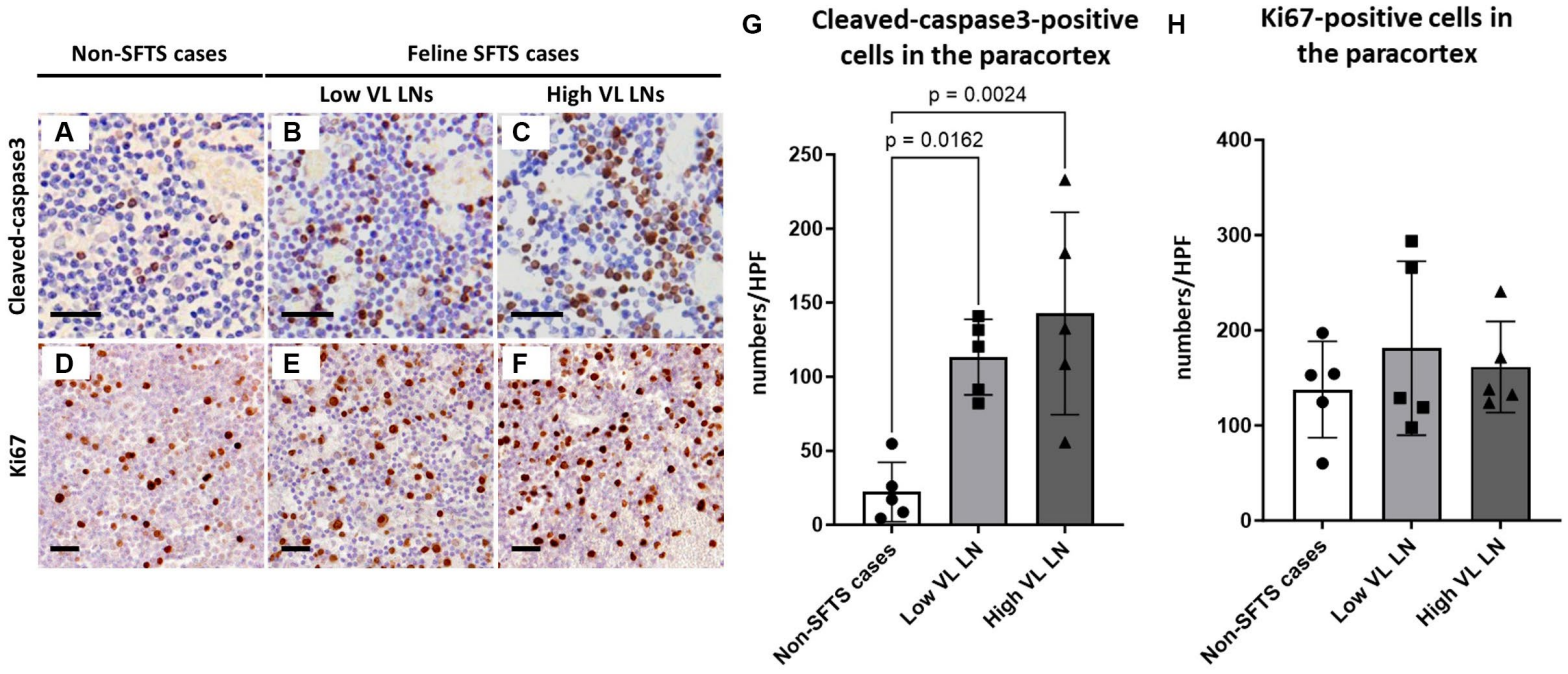
Cleaved-caspase3- and Ki67-positive cells in the paracortex area of the lymph nodes of feline patients with necropsy. Representative results of immunohistochemical analysis of cleaved-caspase3 **(A–C)** and Ki67 **(D–F)**. **(A,D)** Non-severe fever with thrombocytopenia syndrome (SFTS) cases. **(B,E)** Low viral load (VL) lymph nodes (LN); and **(C,F)** High VL LN. Bars indicate 25 μm. **(G,H)** show cleaved-caspase3- and Ki67-positive cell counts, respectively, per high power field of the paracortex area. The bars show the average of five lymph node tissue sections, and each symbol represents the density of each lymph node. Adjusted *p*-values are provided for statistically significant differences.

**Figure 4:**
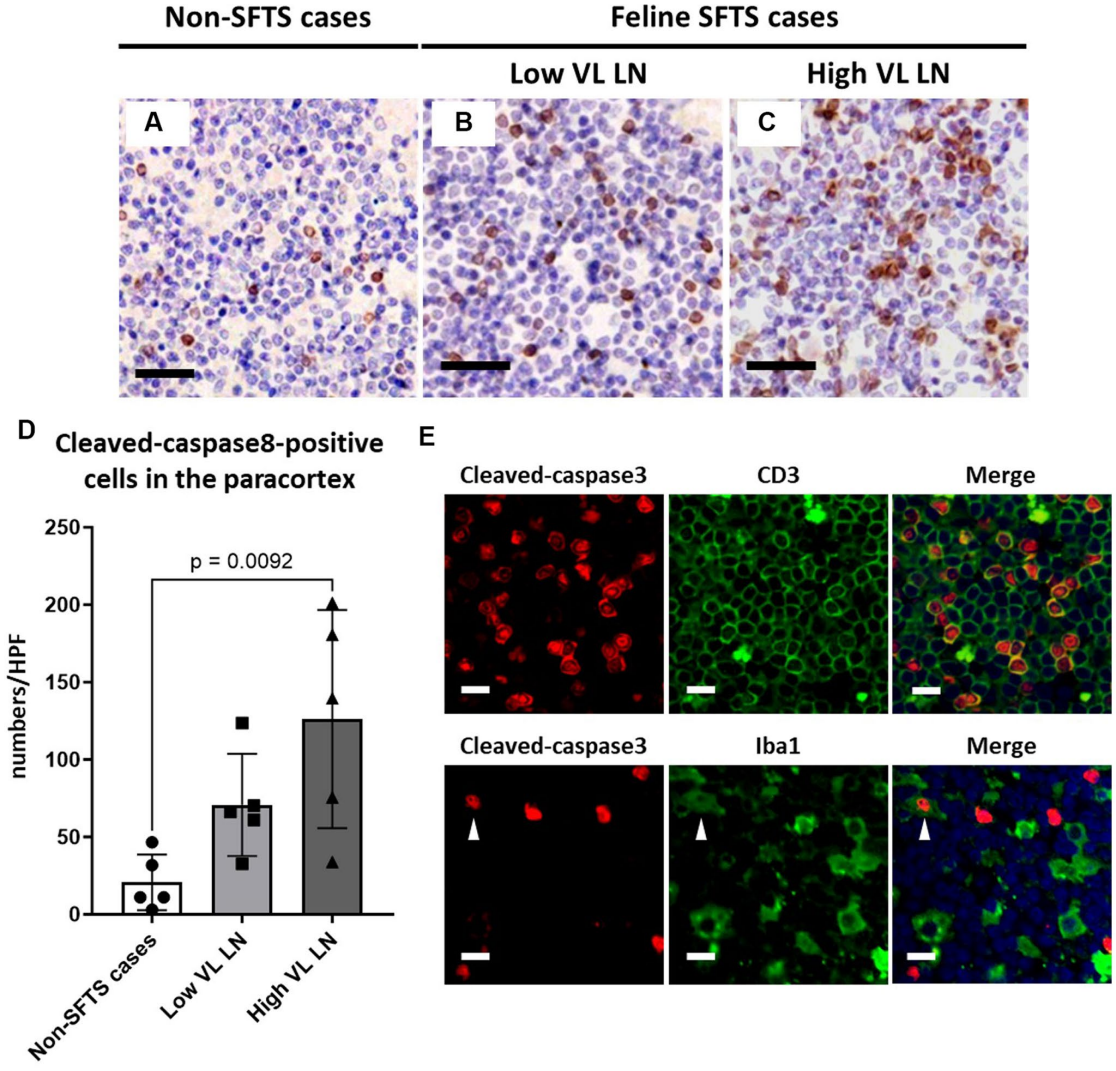
Characterizations of apoptotic cells in the paracortex area of the lymph nodes of necropsy cases. Representative results of immunohistochemical analysis of cleaved-caspase 8 **(A–C)**. **(A)** Non-severe fever with thrombocytopenia syndrome (SFTS) cases. **(B)** Low viral load (VL) lymph nodes (LN), and **(C)** High VL LN. Bars indicate 50 μm. **(D)** Shows cleaved-caspase 8-positive cell counts per high power field of the paracortex area. An adjusted *p*-value is provided for statistically significant difference. **(E)** Shows double staining of cleaved-caspase 3 with CD3 or Iba1 in a SFTSV-low lymph node. White arrows indicate cleaved-caspase 3 and Iba1 double-positive cells. Bars indicate 10 μm.

**Figure 5:**
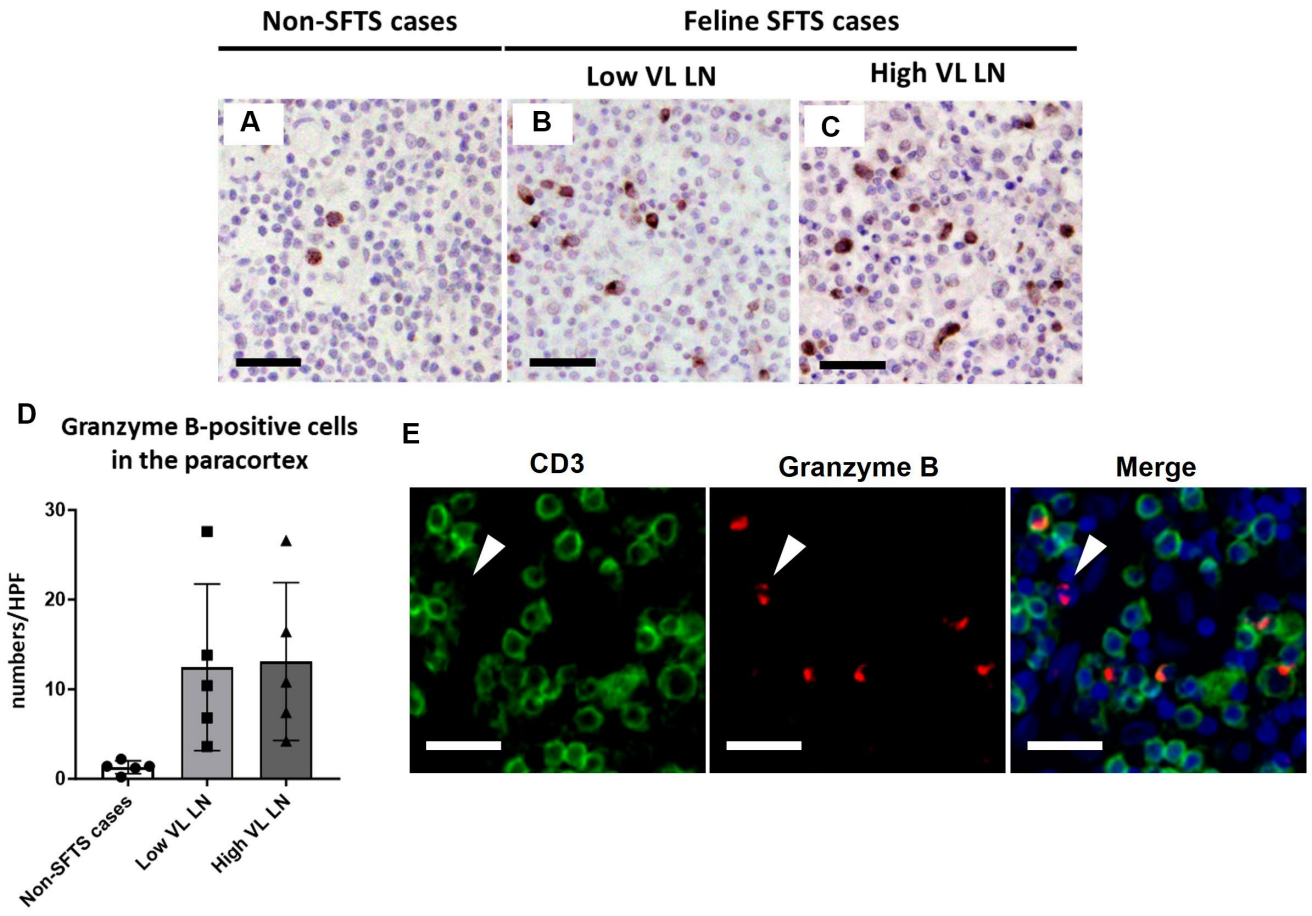
Characterizations of the T-cell population in the paracortex area of the lymph nodes of necropsy cases. Representative results of immunohistochemical analysis of granzyme B **(A–C)**. **(A)** Non-severe fever with thrombocytopenia syndrome (SFTS) cases; **(B)**: Low viral load (VL) lymph nodes (LN), and **(C)** High VL LN. Bars indicate 50 μm. **(D)** Shows granzyme B-positive cell counts per high power field of the paracortex area. **(E)** Shows double staining of CD3 and granzyme B. White arrows indicate granzyme B single positive cells. Bars indicate 25 μm.

Because Tfh and Tfr cells are supplied to lymphoid follicles from the paracortex area, the site of T cell activation and proliferation, defects in paracortical T cells were also suspected. Thus, apoptosis and proliferative activity in the paracortical area were analysed by immunohistochemical detection of cleaved-caspase3 and Ki67. Cleaved-caspase3-positive cells increased by approximately five to six times in feline SFTS cases compared to SFTSV-negative cases (Figures 3A–C,G). In contrast, no obvious changes were observed in the number of Ki67-positive cells (Figures 3D–F,H). Thus, an increase in apoptosis rather than a decrease in proliferation was suggested as the cause of the reduction in the number of Tfh and Tfr cells. Further analysis by cleaved-caspase8 immunohistochemistry was performed to determine whether apoptosis of paracortical lymphocytes was induced by the extrinsic pathway. The number of cleaved-caspase8-positive cells was similar to that of number of cleaved-caspase3-positive cells (Figures 4A–D). These results suggest that the apoptosis of paracortical lymphocytes was mostly induced by the extrinsic pathway. Furthermore, apoptotic cells in the paracortices were characterized by double immunofluorescence staining for cleaved-caspase3 and the T cell marker CD3, or the macrophage marker Iba1. The results showed that most cleaved-caspase3-positive cells were CD3-positive T cells and not Iba1-positive cells (Figure 4E). The cleaved-caspase3-positive rate in CD3-positive cells was approximately 20% in both low viral load and high viral load lymph nodes, while the cleaved-caspase3-positive rate in Iba1-positive cells was higher in high viral load lymph nodes than those in low viral load lymph nodes (Supplementary Figure S3). Furthermore, analysis of granzyme B, a marker of cytotoxic T cells and natural killer cells, was performed to analyse cells with antiviral cell-mediated immune responses, and the results demonstrated an approximately 10-fold increase in SFTSV-infected cases compared to non-SFTS cases (Figures 5A–D). Further characterization of granzyme B-positive cells by double staining with CD3 revealed that most granzyme B-positive cells were CD3-positive T cells (Figure 5E; Supplementary Figure S4).

### Analysis of cats with experimental infection

**Figure 6:**
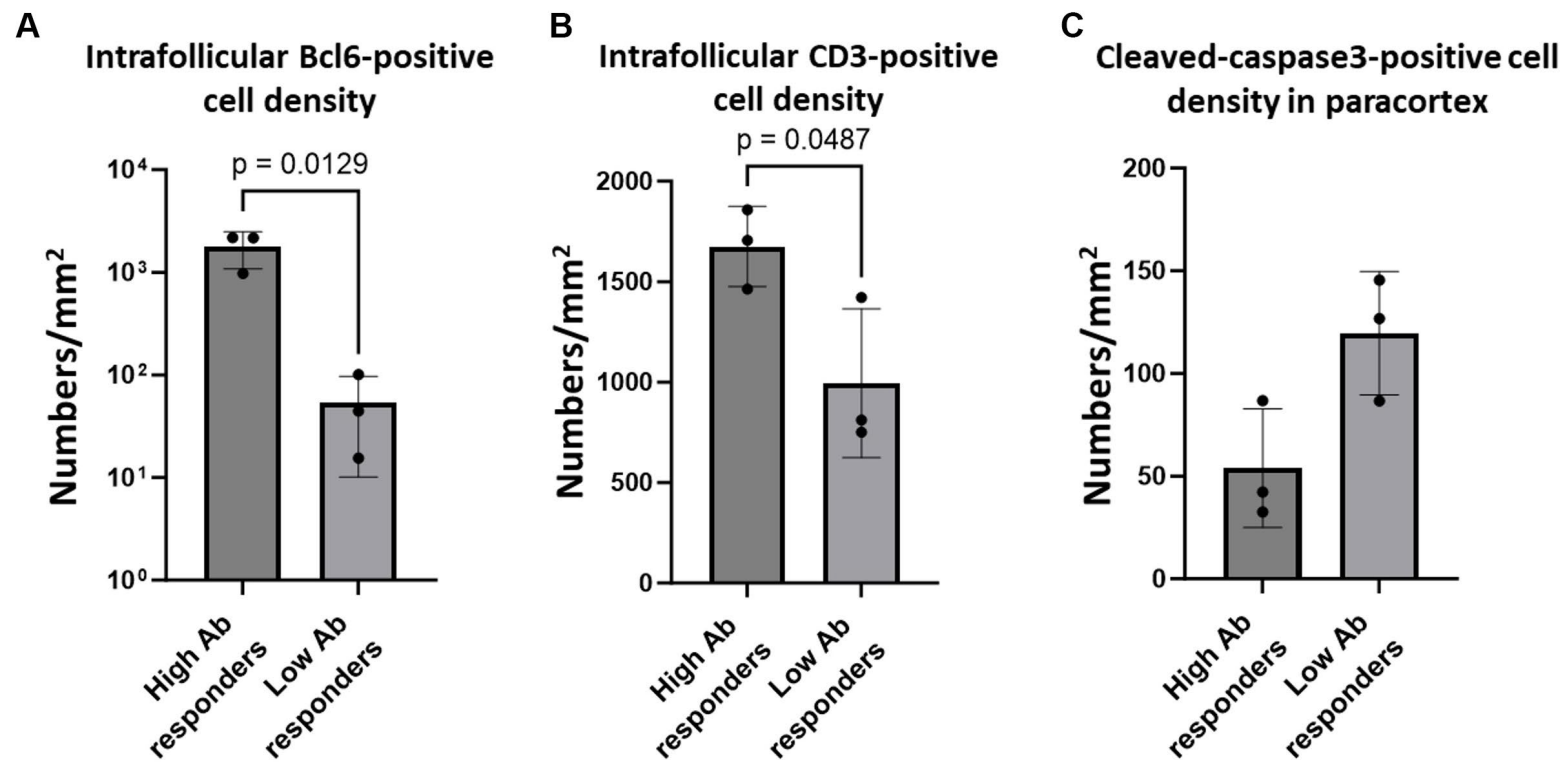
Bcl6-positive germinal centre B cells, intrafollicular CD3-positive cells, and paracortical cleaved-caspase 3-positve cells in the lymph nodes of cats with experimental SFTSV infection. The graphs show the density of intrafollicular Bcl6-positive cells **(A)**, intrafollicular CD3-positive cells **(B)**, and cleaved-caspase 3-positive cells **(C)**. The bars show the average of each group, and each symbol represents the average density of four lymph nodes in each case. Adjusted *p*-values are provided for statistically significant differences.

While our analysis demonstrated an impaired germinal centre response and increased apoptosis of T cells, it remains unclear whether these pathological changes affected the immunological responses in these cats, owing to the lack of information regarding their immunological conditions. To address this, we analysed six cats that were experimentally infected with SFTSV, and their anti-SFTSV antibody titres were examined in a previous study (Park et al., 2019). Among the six cats studied, two (Nos. 1 and 5) showed no obvious clinical signs and had high anti-SFTSV antibody titres (>40). The other three (Nos. 2, 3, and 4) became moribund after SFTSV infection and had only low titres of anti-SFTSV antibodies (<10 or 10), while one cat (No. 6) had a high titre of anti-SFTSV antibody (40) and was moribund (Park et al., 2019). According to these antibody titeres, we grouped Nos. 1, 5, and 6 as high antibody responders, and Nos. 2, 3, and 4 as low antibody responders. Immunohistochemical staining for Bcl6, CD3, and cleaved-caspase3 was performed on submandibular, cervical, axillary, and mesenteric lymph node tissue sections. The viral loads in these lymph nodes are summarized in Supplementary Table S1. The results demonstrated that intrafollicular Bcl6-positive cell densities in high antibody responders were much higher than those in low antibody responders (Figure 6A; Supplementary Figure S6A). Additionally, high antibody responders tended to have higher intrafollicular CD3-positive cell densities and lower paracortical cleaved-caspase3-positive cell densities than those of low antibody responders (Figures 6B,C; Supplementary Figures S6B,C).

## Discussion

In this study, lymphocyte populations in the lymph nodes of cats with SFTS were analysed with a focus on defective germinal centre reactions. The lack of a germinal centre response in feline SFTS cases was first noticed in the histopathological analysis of H&E-stained tissue sections, which was confirmed by immunohistochemical staining of Ki67 and Bcl6 (Figure 1). Because the germinal centre is the site of immunoglobulin class-switch and clonal expansion, our results suggest that class-switch failure and the reduced production of IgG-positive cells found in human patients with SFTS are due to failure of the germinal centre reaction in the lymph nodes. Analysis of experimental infection cases demonstrated that cats with high anti-SFTSV antibody titres contained high numbers of Bcl6-positive cells in their germinal centres (Figure 6A). Importantly, the decrease in the number of Ki67-positive and Bcl6-positive cells had little correlation with the local viral antigen-positive cell number when comparing low SFTSV load lymph nodes and high SFTSV load lymph nodes in SFTS cases (Figure 1). These results suggest that defects in the germinal centre reaction were due to systemic immunological disturbances after SFTSV infection, rather than the direct killing of B cells or plasmablasts by viral infection.

Such severe suppression of the germinal response has been reported in some mouse models of infectious pathogens such as *Salmonella enterica* serovar Typhimurium (Cunningham et al., 2007), *Plasmodium* spp. (Wilmore et al., 2013; Ryg-Cornejo et al., 2016), and *Borrelia burgdorferi* (Hastey et al., 2012). Analysis of these models demonstrated the pivotal contributions of IL-12, TNF-α, and IFN-γ to germinal centre suppression (Ryg-Cornejo et al., 2016; Elsner and Shlomchik, 2019). The cats experimentally infected with SFTSV showed a prominent increase in IL-12 levels from 1 dpi to 7 dpi, while the elevation of TNF-α, and IFN-γ varies even in fatal cases (Park et al., 2019; Matsuu et al., 2023). However, the difference between high Ab responders and low Ab responders were unclear in this observation. In human cases, there is scarce information available about IL-12, whereas elevation of TNF-α and IFN-γ levels is observed in human patients with severe SFTS (Deng et al., 2012; Park et al., 2021). Thus, it is difficult to determine which cytokines contribute to the disruption of the germinal centre reaction and further analysis is required. Histopathological detection of such cytokines will help understand the mechanisms and causative cell subsets of germinal centre disruption.

As one such immunological disturbance, a reduction in the number of follicular T cells was observed in this study (Figures 2, 6B). The decrease in the number of intrafollicular CD3-positive T cells and FoxP3-positive T cells suggested a decline in both Tfh and Tfr cell numbers in cats with SFTS. Because Tfh cells are essential for the germinal centre reaction (Stebegg et al., 2018), a decline in the number of Tfh cells is a possible cause of germinal centre reaction failure. Our findings also demonstrated increased apoptosis of T cells in the paracortex, the site of T cell priming and the source of follicular and peripheral functional T cells (Figures 3, 4E, 6C). A decline in T cell numbers has also been observed in the peripheral blood of patients with severe SFTS (Sun et al., 2014; Lu et al., 2015; Liu et al., 2017; Li M. M. et al., 2018), and Fas/FasL-mediated apoptosis has been suggested as a cause of this decline (Li M. M. et al., 2018). Consistently, the results of cleaved-caspase8 analysis suggests that apoptosis of T cells in the lymph nodes of feline SFTS cases was also due to the activation of the extrinsic pathway (Figures 4A–D), which is mediated by the engagement of death receptors with death ligands, followed by the cleavage of caspase8 (Elmore, 2007). The loss of T cells has also been observed in various acute life-threatening viral infections, and the contribution of Fas/FasL and cytokines, such as TNFα, IL-6, IFN-γ, and IL-10, has been suspected (Guo et al., 2021). Although our study demonstrated that most of cleaved-caspase3-positive cells express Fas (Supplementary Figure S5), the role of the Fas/FasL system could not be elucidated because the increase in FasL expression levels and the contribution of other cytokines could not be analysed in this study. Further studies on lymph node and bone marrow sections will provide insights into the mechanism underlying lymphocytopenia in SFTS. Despite the increased apoptosis of T cells, the number of granzyme B-positive cells increased in SFTSV-infected cases, and most of these cells were cytotoxic T cells (Figure 5). Previous studies analysing peripheral blood leukocyte of human patients with SFTS showed a decreasing tendency for CD8-positive cytotoxic T cells (Sun et al., 2014; Lu et al., 2015; Liu et al., 2017). The clear tendency for an increase in the number of lymph node cytotoxic T cells found in this study may indicate that the lymph nodes are the major site of viral replication and that Th cells were more susceptible to unknown apoptosis-inducing stimuli under SFTS conditions. In this study, we reported the pathological changes in the lymph node as one cause of immunological disturbance in severe SFTS. However, germinal cecntre failure and T-cell apoptosis in the lymph node are probably just a part of the immunological disturbance in severe SFTS patients. For example, splenic lymphocyte of severe feline SFTS patients usually show massive necrosis due to more rapid disease progression than lymph node (Sakai et al., 2021), and necrosis of splenic lymphocyte also presumed to contribute to lymphocytopenia. Furthermore, hemophagocytosis, that is commonly found in feline SFTS cases and often reported in human SFTS cases (Weng et al., 2014; Kato et al., 2016 Shin et al., 2016 Sakai et al., 2021), may exacerbate decrease of lymphocyte by phagocytosing lymphocyte. For understanding whole immunopathology of SFTS, analyses focusing on individual phenomena are further required.

## Data availability statement

The raw data supporting the conclusions of this article will be made available by the authors, without undue reservation.

## Ethics statement

The animal studies were approved by Institutional Animal Care and Use Committee of the NIID. The studies were conducted in accordance with the local legislation and institutional requirements. Written informed consent was obtained from the owners for the participation of their animals in this study.

## Author contributions

YS: Conceptualization, Data curation, Funding acquisition, Investigation, Methodology, Resources, Visualization, Writing – original draft, Writing – review & editing. SMu: Conceptualization, Investigation, Methodology, Resources, Visualization, Writing – original draft. YK: Investigation, Resources, Writing – review & editing. SK: Resources, Writing – review & editing. MSak: Methodology, Resources, Supervision, Writing – review & editing. MM: Conceptualization, Project administration, Resources, Supervision, Writing – review & editing. E-sP: Investigation, Resources, Supervision, Writing – review & editing. MSh: Investigation, Writing – review & editing. NN: Investigation, Resources, Writing – review & editing. YA: Investigation, Writing – review & editing. TY: Investigation, Writing – review & editing. NI-Y: Investigation, Resources, Writing – review & editing. SF: Investigation, Writing – review & editing. SW: Investigation, Writing – review & editing. TK: Investigation, Writing – review & editing. AO: Investigation, Writing – review & editing. MK: Investigation, Writing – review & editing. KI: Investigation, Writing – review & editing. MSai: Resources, Writing – review & editing. SMo: Resources, Supervision, Writing – review & editing. TS: Conceptualization, Project administration, Supervision, Writing – review & editing. KM: Conceptualization, Project administration, Resources, Supervision, Writing – review & editing.
