# Supplementary material for: Lethal severe fever with thrombocytopenia syndrome virus infection causes systemic germinal centre failure and massive T cell apoptosis in cats
Source: Front Microbiol. 2024 Jan 5;14:1333946. doi: 10.3389/fmicb.2023.1333946 (PMC10796997; doi:10.3389/fmicb.2023.1333946)
Supplement: Supplementary file 1 [file Presentation_1.PDF]

Supplementary materials

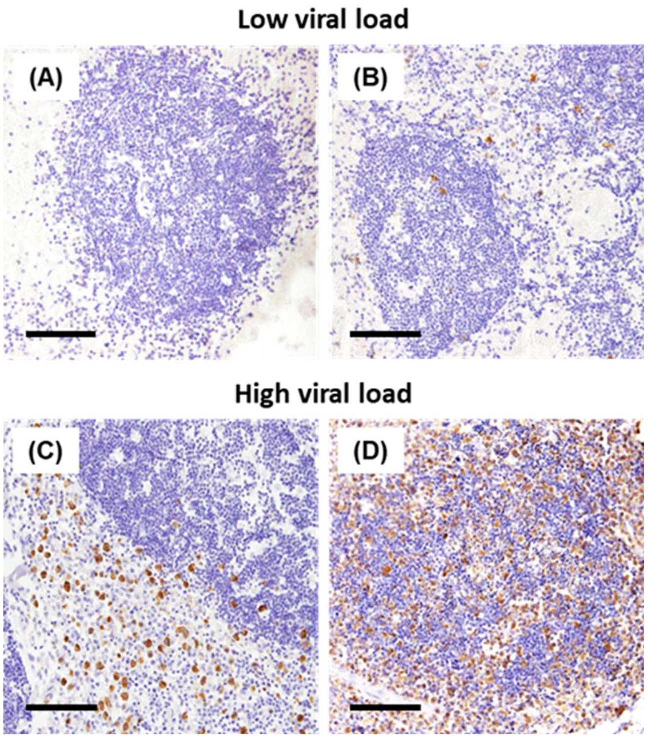

Supplementary Figure 1. Grading of lymph nodes from SFTSV-infected cats. (A) and (B) Low viral load lymph nodes. The lymph nodes contain no or few SFTSV-positive cells. (C) and (D) High viral load lymph nodes. Significant to massive number of SFTSV-positive cells were found in the cortex area. Bars indicate 200 μm.

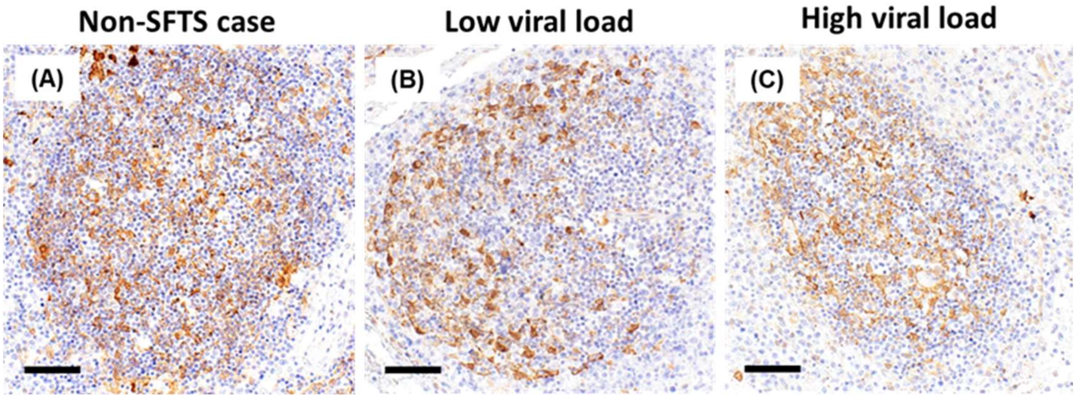

Supplementary Figure 2. Confirmation of follicular structure with immunohistochemistry against fascin. The representative results of the lymph node from non-SFTS cases (A), low viral load lymph node (B), high viral load lymph node (C). Bars indicate 200 µm.

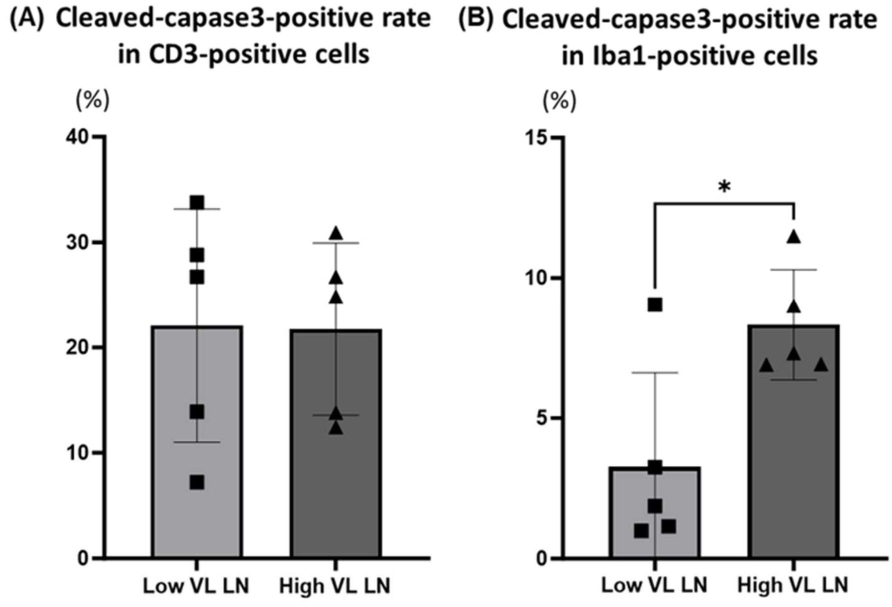

Supplementary Figure 3. Cleaved-caspase 3-positive cell rate in CD3-positive cells (A) and Iba1-positive cells (B) were calculated using photos of double-labelling immunofluorescence. The bars show the average of five lymph nodes in each group: low viral load (VL) lymph node (LN) and high VL LN. Each symbol represents the rate of each lymph nodes. The significance between groups was examined using Student's t-test and * indicates $p < 0.05$.

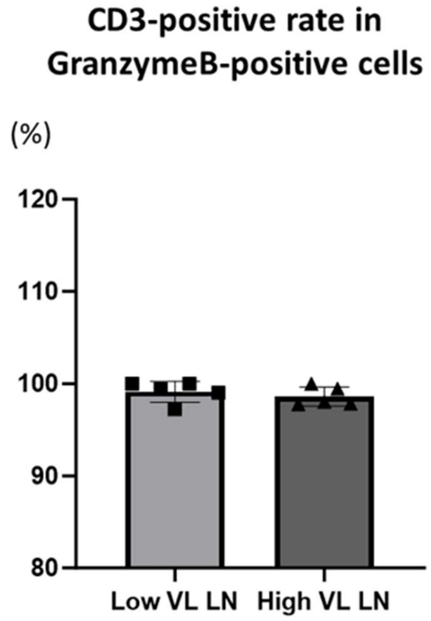

Supplementary Figure 4. CD3- positive cell rate in granzyme B-positive cells was calculated using photos of double-labelling immunofluorescence. The bars show the average of five lymph nodes in two groups: low viral load (VL) lymph node (LN) and high VL LN. Each symbol represents the rate of each lymph node. The significance between groups was examined using Student's t-test and confirmed as not significant.

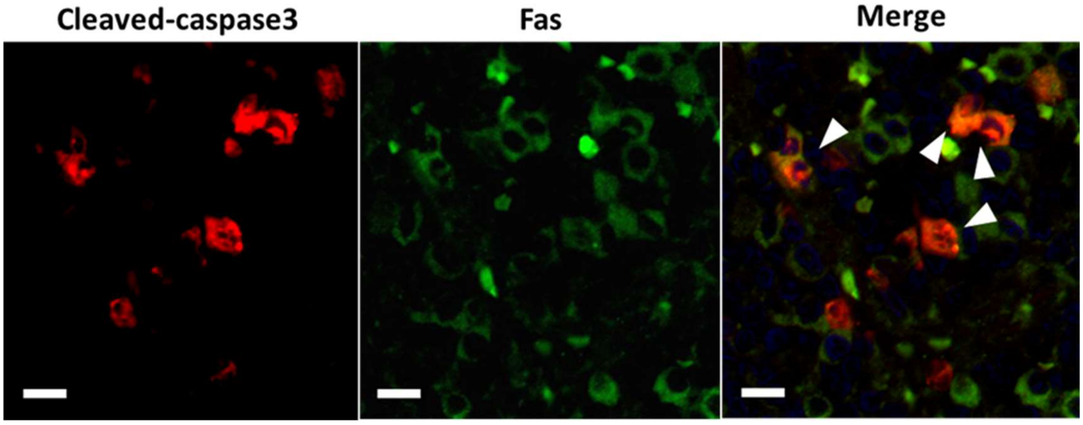

Supplementary Figure 5. Double-labelling immunofluorescence analysis of cleaved-caspase 3 and Fas in a SFTSV-low lymph node. White arrows indicate double-positive cells. Bars indicate 10 µm.

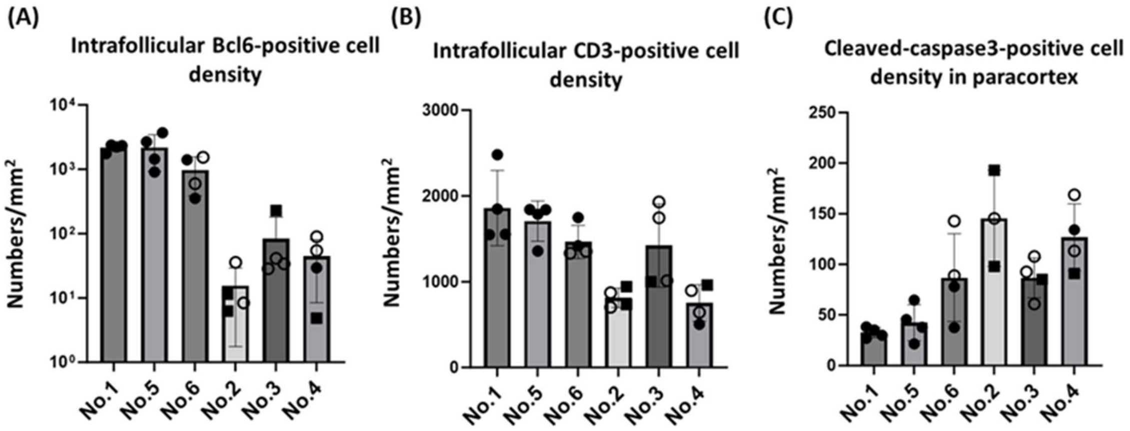

Supplementary Figure 6. Bcl6-positive germinal centre B cells, intrafollicular CD3-positive cells, and paracortical cleaved-caspase 3-positve cells in the lymph nodes of cats with experimental SFTSV infection. The graphs show the density of intrafollicular Bcl6-positive cells (A), intrafollicular CD3-positive cells (B), and cleaved-caspase 3-positive cells. The bars show the average of four lymph node tissue sections and each symbol represents the density of each lymph node. Black circles, white circles, and squares indicate lymph nodes with no SFTSV-positive cells, low viral load, and high viral load, respectively.

Supplemental table 1. Viral load in the lymph node of the cat with experimental infection

Antibody response	I.D.	SFTSV viral load			
		Submandibular	Cervical	Axillary	Mesenteric
High reponders	No.1	N.D.	N.D.	N.D.	N.D.
	No.5	N.D.	N.D.	N.D.	N.D.
	No.6	Low	Low	N.D.	N.D.
Low responders	No.2	High	High	Low	Low
	No.3	Low	Low	High	Low
	No.4	Low	Low	N.D.	High

N.D.; Not detected
